# The role of zinc in the anti-tumour and anti-cachectic activity of D-myo-inositol 1,2,6-triphosphate

**DOI:** 10.1038/sj.bjc.6605562

**Published:** 2010-02-09

**Authors:** S T Russell, P M A Siren, M J Siren, M J Tisdale

**Affiliations:** 1 Nutritional Biomedicine, School of Life and Health Sciences, Aston University, Birmingham B4 7ET, UK; 2 Bioneris Ab, IAM, Adolf Fredriks Kyrkogata 13, Stockholm 11137, Sweden; 3 JGK Memorial Research Library and Laboratory, Helsinki Töölön k 1900260, Finland

**Keywords:** cachexia, tumour growth, zinc, *α*-trinositol

## Abstract

**Background:**

D-myo-inositol-1,2,6-triphosphate (*α*-trinositol, AT) is a polyanionic molecule capable of chelating divalent metal ions with anti-tumour and anti-cachectic activity in a murine model.

**Methods:**

To investigate the role of zinc in this process, mice bearing cachexia-inducing MAC16 tumour were treated with AT, with or without concomitant administration of ZnSO_4_.

**Results:**

At a dose of 40 mg kg^−1^, AT effectively attenuated both weight loss and growth of the MAC16 tumour, and both effects were attenuated by co-administration of Zn^2+^. The concentration of zinc in gastrocnemius muscle increased with increasing weight loss, whereas administration of AT decreased the levels of zinc in plasma, skeletal muscle and tumour, which were restored back to control values after administration of ZnSO_4_.

**Conclusion:**

These results suggest that zinc is important in both tumour growth and cachexia in this animal model.

Trace metals such as zinc are essential components of many enzymes and transcription factors. Deficiency of zinc results in reduced food intake and growth, impaired synthesis of DNA and dysfunction of the immune system (MacDonald, 2000). Zinc has been shown to be involved in intracellular signalling events (Yamasaki *et al*, 2007), whereas an extracellular zinc-sensing receptor, not requiring zinc influx, has been shown to trigger the release of Ca^2+^ from intracellular stores (Hershfinkel *et al*, 2001). Although zinc is an essential trace element, high concentrations are toxic to cells, and zinc uptake, intracellular storage and efflux are carefully maintained (Kim *et al*, 2004).

There is evidence for aberrant zinc transport into tumour cells, and it has been suggested that zinc availability may be essential for tumour growth (Lee *et al*, 2003). Overexpression of the zinc transporter, ZIP4, compared with the surrounding normal tissue, is seen in 94% of clinical specimens of pancreatic adenocarcinoma, suggesting that it may contribute to the pathogenesis and progression of the disease (Li *et al*, 2007). An increased expression of ZIP4 in pancreatic cancer cells increased intracellular zinc concentration, cell proliferation and tumour volume in nude mice. Zinc has also been shown to induce a dose-dependent increase in proliferation of the human prostate adenocarcinoma cell line, PC-3, and this was attenuated by the zinc chelator Ca EDTA (Dubi *et al*, 2008). Extracellular zinc also attenuated cell death. These results suggest that zinc chelation may be an effective measure to inhibit tumour growth.

There are also changes in zinc concentration in tumour and skeletal muscle of rats bearing a methylcholanthrene-induced fibrosarcoma during muscle wasting (Larsson *et al*, 1987). Thus, at 12 days after tumour transplantation, when cachexia is maximal, there is a significant decrease in serum zinc concentration, with a corresponding increase in both tumour and skeletal muscle. A progressive decrease in plasma zinc was also observed during growth of rat adenocarcinoma (Philcox *et al*, 1994). These results suggest that zinc may also have a role in the process of atrophy of skeletal muscle.

We have previously shown (Russell *et al*, 2009) that the polyanionic compound D-myo-inositol 1,2,6-triphosphate (*α*-trinositol, AT) attenuated both tumour growth and muscle atrophy in mice bearing the cachexia-inducing MAC16 tumour. *α*-Trinositol has been shown to chelate divalent metal ions such as Ca^2+^ and Zn^2+^, which bind to phosphates P1 and P6 of the inositol ring structure (Falemez and Speiss, 2001), but there have been no studies on whether this is important in the anti-cachectic and anti-tumour effect of AT. We hypothesised (PS and MJS) that zinc has an important role in the onset and progression of cancer cachexia. The current study investigates the role of zinc in the biological effects of AT in mice bearing the MAC16 tumour and its relationship to tumour growth and cachexia

## Materials and methods

### Materials

D-myo-inositol 1,2,6-triphosphate (AT) was supplied by JGK Memorial Research Library and Laboratory (Helsinki, Finland)/Bioneris Ab (Stockholm, Sweden).

### Animals

The MAC16 tumour was passaged in pure strain male NMRI mice (average weight 25 g), which were obtained from our own inbred colony, and were fed a rat and mouse breeding diet (Special Diet Services, Witham, UK) and water *ad libitum*. Tumour fragments were obtained from donor animals selected as those having the maximum weight loss, and were implanted s.c. into the flank by means of a trochar, as previously described (Bibby *et al*, 1987). Weight loss was evident from 10 days after tumour transplantation and animals were entered into the study when they had lost ∼5% of their starting body weight. Animals were randomised into groups of five to receive either solvent (PBS), AT (40 mg kg^−1^) or AT (40 mg kg^−1^) plus 8.05 *μ*g ZnSO_4_ in 25 *μ*l PBS administered i.v. 1 h after AT. A fourth group received ZnSO_4_ alone. Both PBS and AT were administered s.c. three times a day. Tumour volume, body weight and food and water intake were monitored daily. Animals were killed by cardiac puncture under terminal anaesthesia when the body weight loss was at, or before, 20%, as approved by the British Home Office. The ethical guidelines that were followed met the standards required by the UKCCR guidelines (Workman *et al*, 1998). Both tumour and gastrocnemius muscles were removed, snap frozen in liquid nitrogen and stored at −80°C until measurement of zinc concentration.

### Measurement of zinc concentrations

Tissues were weighed in crucibles, which were previously washed in 5% Lipsol, and rinsed with deionised bio-filtered water, followed by trace analysis using nitric acid. The crucibles were placed in a furnace at room temperature, and the temperature was increased to 150°C for 1 h, then 600°C for 2 h, followed by 800°C overnight. A fine white ash, free from organic material, was obtained, and the crucibles were cooled and stored in desiccators. The crucibles were subsequently re-weighed to obtain the mass of ash, which was dissolved in 10 ml of trace element analysis concentrated nitric acid and sonicated for 30 min to ensure stabilisation. The dissolved ash was then transferred to a 100 ml volumetric flask, which was made up to volume with deionised bio-filtered water. The sample was then analysed for zinc using a PerkinElmer Analyst 100 atomic absorption spectrophotometer (PerkinElmer, Waltham, MA, USA), at a wavelength of 213.9 nM, slit 0.7 mm, using air/acetylene gas and a lamp current of 10 mA. Standards of known concentrations were used to construct calibration curves from which the concentration of zinc in the sample could be determined.

### Statistical analysis

Results are presented as mean±s.e.m. Differences in means between groups were determined by one-way analysis of variance (ANOVA), followed by the Tukey–Kramer multiple comparison test. *P*-values <0.05 were considered significant.

## Results

**Figure 1:**
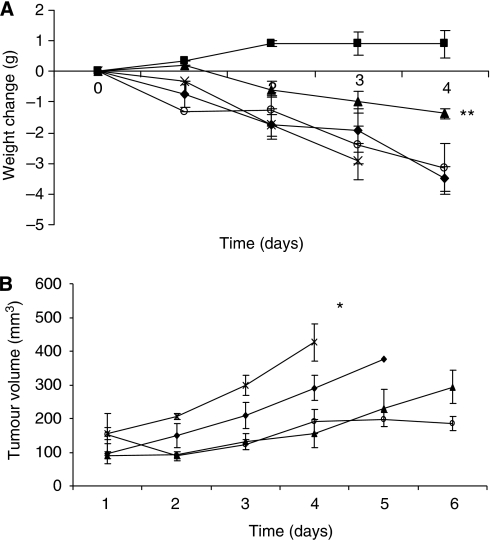
Weight change (**A**) and tumour volume (**B**) in either non-tumour-bearing mice (▪) or in mice bearing the MAC16 tumour treated with either PBS (⧫), AT (▴), AT +Zn^2+^ (X) or Zn^2+^ (O) at the doses and schedule as detailed in Materials and methods. Differences from PBS-treated animals are indicated as ^*^P<0.01 or ^**^P<0.001.

The effect of AT on body weight of mice bearing the MAC16 tumour is shown in Figure 1A. A dose of 40 mg kg^−1^ of AT was chosen, as this has been shown to produce optimal inhibition of both weight loss and tumour volume (Russell *et al*, 2009). This study again showed that AT attenuated the loss of body weight produced by the MAC16 tumour, and this was completely reversed by administration of Zn^2+^ 1 h after AT. Administration of Zn^2+^ alone had no effect on weight loss compared with that of PBS controls. A similar result was obtained with regard to tumour volume (Figure 1B). Thus, AT also attenuated the tumour growth rate, and this was completely reversed by co-administration of Zn^2+^. However, Zn^2+^ alone inhibited tumour growth rate to the same extent as AT. These results show the importance of Zn^2+^ in the anti-cachectic and anti-tumour activity of AT.

**Figure 2:**
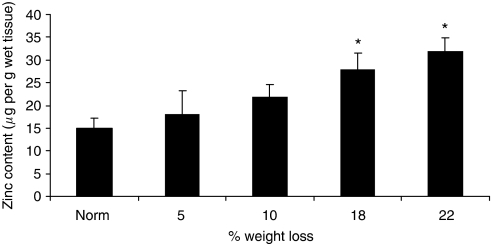
Zinc concentrations in gastrocnemius muscle of non-tumour-bearing mice and mice bearing the MAC16 tumour and with different extents of weight loss. The results are expressed relative to non-tumour-bearing animals after allowance for different muscle weights. Differences from non-tumour-bearing animals are shown as ^*^P<0.05.

**Figure 3:**
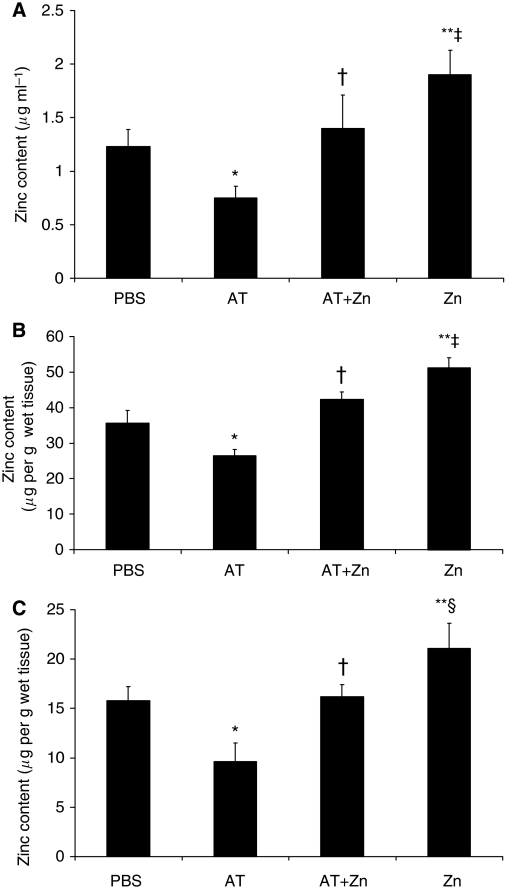
Concentration of zinc in plasma (**A**), gastrocnemius muscle (**B**) and tumour (**C**) of mice bearing the MAC16 tumour after 4 days of treatment with PBS, AT, AT plus Zn^2+^ or Zn^2+^ alone, as depicted in Figure 1. Differences from PBS controls are shown as ^*^P<0.05 or ^**^P<0.01, whereas differences from AT are shown as ^†^*P*<0.05 and differences from AT+Zn as ^‡^*P*<0.05 or ^§^*P*<0.01.

As previously reported in weight-losing rats bearing a fibrosarcoma (Larsson *et al*, 1987), there was an increase in zinc concentration in the gastrocnemius muscle of mice bearing the MAC16 tumour, which became significant when weight loss was <18% (Figure 2). There was no difference in the zinc concentration of plasma between tumour-bearing and non-tumour-bearing animals (Figure 3A). The reason for the accumulation of zinc in skeletal muscle is not known. However, treatment of mice bearing the MAC16 tumour with AT caused a significant reduction in zinc concentration in the plasma (Figure 3A), gastrocnemius muscle (Figure 3B) and tumour (Figure 3C), when measured after 4 days of treatment, as shown in Figure 1. Administration of Zn^2+^ to animals receiving AT increased the levels of Zn^2+^ in plasma, gastrocnemius muscle and tumour up to the levels found in PBS controls (Figure 3), consistent with the ability of Zn^2+^ to attenuate the action of AT on weight loss (Figure 1A) and tumour volume (Figure 1B). Administration of Zn^2+^ in the absence of AT caused a significant increase in the levels of Zn^2+^ in plasma, gastrocnemius muscle and tumour (Figure 3), consistent with an increase in weight loss and tumour volume (Figure 1). These results suggest that both tumour growth and weight loss may be limited by the availability of Zn^2+^.

## Discussion

The results of this study show the importance of Zn^2+^ in the process of both muscle wasting and tumour growth in mice bearing the MAC16 tumour. There have been few studies on the role of Zn^2+^ in skeletal muscle atrophy, although an increase in Zn^2+^ has been observed in the skeletal muscle of rats during the progress of cachexia (Larsson *et al*, 1987), as also observed in this study in mice bearing the MAC16 tumour. A similar result was obtained in mice transplanted with the Lewis Lung Carcinoma, which also produces cachexia (Frank *et al*, 1986). In this study, the zinc concentration in muscle was three times that of normal control, and there was also a re-distribution to the bone. One mechanism by which Zn^2+^ could participate in muscle atrophy is through activation of nuclear factor-*κ*B (NF-*κ*B) through phosphorylation of p65/Rel A at multiple serine residues (Kim *et al*, 2007). Activation of NF-*κ*B in the muscle of mice has been shown to lead to atrophy through increased expression of proteasome subunits and the E3 ligase, MURF1 (Cai *et al*, 2004). It occurs through activation of caspases-3 and -8, and the subsequent autophosphorylation of the dsRNA-dependent protein kinase (PKR) (Suen *et al*, 2003). Autophosphorylation of PKR in skeletal muscle has been shown to inhibit protein synthesis owing to phosphorylation of eukaryotic initiation factor-2 (eIF2) on the *α*-subunit, and increased protein degradation through the NF-*κ*B-mediated induction of the ubiquitin–proteasome pathway (Eley and Tisdale, 2007). The activity of caspases-3 and -8, phosphorylation of PKR and eIF2*α* and the activity and expression of the ubiquitin–proteasome pathway have been shown to be attenuated by AT in mice bearing the MAC16 tumour (Russell *et al*, 2009).

These results provide some evidence that the attenuation of loss of skeletal muscle by AT is separate from its anti-tumour activity, although both effects seem to be due to its ability to chelate Zn^2+^. Studies *in vitro* also support a separate role of AT in attenuating muscle atrophy. Thus, using murine myotubes as a model of skeletal muscle, AT has been shown to attenuate both the induction of protein degradation and depression of protein synthesis induced by proteolysis-inducing factor, angiotensin II, lipopolysaccharide and TNF-*α* (Russell *et al*, 2009 and unpublished results), and these effects could be reversed by addition of Zn^2+^. These studies also suggest that Zn^2+^ is required for the caspase-3/-8-mediated activation of PKR. As AT is highly negatively charged, it is unlikely that it would readily penetrate into cells; hence, it is likely that the effects are mediated through the chelation of extracellular Zn^2+^.

In view of the potential mechanism by which AT attenuates tumour growth rate by chelation of Zn^2+^, and the role of zinc in the proliferation of pancreatic cancer (Li *et al*, 2007), it was surprising that administration of Zn^2+^ alone attenuated tumour growth rate to the same extent as AT. However, it is known that although tumours need Zn^2+^ to grow and survive, excess Zn may induce apoptosis (Murakami and Hirano, 2008). Extracellular zinc has been shown to regulate growth and survival of prostate cancer cells through a putative zinc-sensing receptor (Dubi *et al*, 2008). Low concentrations of Zn^2+^ were shown to stimulate cell proliferation through activation of the mitogen-activated protein kinase and phosphatidylinositol-3-kinase pathways mediated by a Gq-coupled receptor. (Azriel-Tamir *et al*, 2004) However, high concentrations of Zn^2+^ led to desensitisation of the zinc receptor possibly through constitutive desensitisation, as for other G-protein-coupled receptors, and through inhibition of tumour growth. These results could explain the apparent anomaly that both chelators of Zn^2+^ and Zn^2+^ itself inhibit tumour growth.

These results suggest that AT is a novel agent with anti-cachectic and anti-tumour activity due to its ability to chelate Zn^2+^. Further studies are required on the role of Zn^2+^ in the signalling cascade leading to muscle atrophy, in order to elucidate the mode of action of this important metal in relation to cachexia.
